# Verification of dried blood spot as a sample type for HIV viral load and early infant diagnosis on Hologic Panther in Zambia

**DOI:** 10.1186/s13104-023-06344-9

**Published:** 2023-05-11

**Authors:** Precious Simushi, Mukoshya Nchima Kalunga, Tuku Mwakyoma, Mulenga Mwewa, Lweendo Muchaili, Nchimunya Hazeemba, Chileshe Mulenga, Patience Mwewa, Kaseya O. R. Chiyenu, John Kachimba, Powell Choonga, Aaron Shibemba, Benison M. Hamooya, Mowa Zambwe, Peter J. Chipimo, Lackson Kasonka

**Affiliations:** 1 Livingstone University Teaching Hospital Laboratory, Livingstone, Zambia; 2 Ministry of Health, Ndeke House, Lusaka, Zambia; 3 University Teaching Hospital, Pathology, Lusaka, Zambia; 4 Mulungushi University, Livingstone, Zambia; 5 Benefits Department, Workers Compensation Fund Control Board, Lusaka, Zambia; 6 World Health Organization, Lusaka, Zambia; 7 Ministry of Health, Lusaka, Zambia

**Keywords:** Dried blood spot, Early infant diagnosis, HIV viral load, Hologic Panther

## Abstract

**Objective:**

Zambia has embarked on improving the diagnostic capacity by setting up high throughput and accurate machines in the testing process and introduction of dried blood spot (DBS) as a sample type. This was a cross sectional study to verify dried blood spot as a sample type for HIV viral load and early infant diagnosis (EID) on Hologic Panther platform and Evaluate the analytical performance (precision, linearity and measurement of uncertainty) of the Hologic Panther.

**Results:**

The specificity and sensitivity of EID performance of Aptima Quant Dx assay on Hologic panther machine against the gold standard machine COBAS Taqman (CAP/CTM) was 100% with an overall agreement of 100%. The quantitative HIV Viral Load (VL) accuracy had a positive correlation of (0.96) obtained against the gold standard (plasma samples) run on COBAS4800 platform. Analytical performance of the Hologic panther machine was evaluated; Precision low positive repeatability 3.50154 and within lab 2.268915 at mean 2.88 concentration and precision high positive repeatability 1.116955 and within lab 2.010677 at mean 5.09 concentration were obtained confirming manufacturers claims. Uncertainty of measurement for this study was found to be ± 71 copies/ml. Linearity studies were determined and all points were within acceptable limits. We therefore recommend DBS as a sample type alternative to plasma for the estimation of HIV-1 viral load and EID diagnosis on the Hologic panther machine.

## Introduction

Zambia is part of the Joint United Nations Programme on HIV/AIDS (UNAIDS) targets of 95–95–95 which means: 95% of all HIV-positive individuals should be diagnosed, provide antiretroviral therapy (ART) for 95% of those diagnosed and achieve viral suppression for 95% of those treated [1]. However, sub Saharan countries have had challenges in achieving the third 95 goal partly as a result of Covid-19 pandemic [2]. In order to achieve this goal, Zambia has embarked on improving the diagnostic capacity by setting up high throughput and accurate machines in the testing process and by introducing dried blood spot (DBS) as a sample type on different PCR platforms across the country although DBS as a sample type has been already in use for early infant diagnosis (EID) in most resource limited countries [3] and it has been observed that the use of DBS can be a practical tool to mitigate challenges to deal with transport of blood specimens, trained phlebotomist, centrifugation and ultimately a well functional cold chain [4].

World Health Organization (WHO) consolidated guidelines for HIV care and treatment indicate that viral load testing is a preferred option for monitoring clients on antiretroviral therapy [5]. Prompt testing ensures timely monitoring, treatment adherence and efficacy, and minimizing treatment failure [6]. It is recommended that routine monitoring of antiretroviral therapy (ART) clients using viral load (VL) plasma testing be done at 6 months and every 12 months [7]. However, in Zambia, there is low coverage of viral load testing [8]. This is because of hard to reach areas, inadequate resources for immediate transportation of the specimens, storage and maintaining sample integrity as in the case of plasma, equipment breakdown, and stock outs of reagents at testing sites [7, 8, 8–13]. Therefore, the introduction of DBS as a sample type for VL and EID on a new platform Hologic Panther might increase the service delivery and mitigate some of these issues to do with costs, storage and transport requirements.

DBS viral load measurement has proven to be a better alternative to plasma specimens, more especially in remote settings with no laboratories [14–19]. The monitoring of clients on ART with HIV VL as recommended in developed countries has proven to be a challenge in resource limited countries this can be attributed to high costs and stringent storage and transport requirements that comes with plasma as a sample type [15]. The use of DBS for both HIV VL and EID of HIV-1 exposed infants enables easy access to treatment, timely initiation of antiretroviral therapy (ART), thereby allowing early diagnosis and treatment to slow disease progression and reduce mortality [4, 20–22]. It also simplifies virological monitoring of ART clients in resource limited settings [15]. The addition of a high through put machine Hologic Panther platform and use of the Aptima Quant Dx HIV-1 assay for both diagnosis and quantification to the testing services will improve Turn-around time, mitigating the low to medium throughput technology which can pose as a hindrance to early treatment [23].

## Main text

### Methods

#### Study design and setting

We conducted a retrospective laboratory based cross-sectional study at the Livingstone University Teaching Hospital laboratory (LUTH). The LUTH laboratory is located in Livingstone town, southern part of Zambia. It is a main referral center for specialized testing for HIV VL and EID diagnosis servicing 13 districts in the province.

#### Sample size

- Qualitative:
  120 samples (60 positives and 60 negatives) of less than or equal to 18 months were used (as per manufactures recommendation)
- Quantitative:
  HIV VL 154 samples
  Open-Epi sample size calculator was used with 11.3% HIV prevalence in Zambia at 5% CI

EID DBS cards collected from different facilities that had been received at Livingstone laboratory for routine testing were conveniently sampled after being confirmed reactive and non-reactive on current platform COBAS Taqman (CAP/CTM) from infants of age ≤ 18 months. A sample size of 60 DBS cards with at least 2 spots (as per manufacturer recommendation) were retrieved from storage within a retention period of less than 18 months. For HIV VL, we conveniently sampled 154 HIV VL stored samples comprising of three levels; Target not detected, below titer and above titer. These were in EDTA containers collected within 24 h and spotted on DBS cards and left to dry for 24 h before processing on the Hologic Panther machine. We used results generated by COBAS 4800 to assign the levels.

#### Test methods

The COBAS Taqman (CAP/CTM) HIV-1 qual test is a qualitative nucleic acid amplification test for the detection of HIV type 1 RNA and proviral DNA in plasma, anti-coagulated fresh whole blood and DBS. The test is based on four major processes; Sample preparation and incubation, Sample preparation to isolate HIV-1 target nucleic acids-both processes utilizing the magnetic glass particle technology, Reverse transcription of the target RNA to generate complimentary DNA(cDNA) and Detection of cleaved dual labelled oligonucleotide detection probe specific to the target.

The COBAS4800 HIV-1 is based on fully automated sample preparation followed by PCR amplification and detection. The cobas^®^ 4800 System consists of the cobas × 480 sample preparation instrument and the cobas z 480 real-time PCR analyzer. Real-time detection and discrimination of PCR products is accomplished by measuring the fluorescence of the released reporter dyes for the viral targets and RNA QS, respectively.

The Aptima HIV-1 Quant Dx assay involves three main steps, which all take place in a single tube on the Panther system: these include target capture, target amplification by transcription-mediated amplification (TMA), and detection of the amplification products (amplicon) by the fluorescent labeled probes (torches).

### Statistical analysis

The collected data were entered in excel, thereafter exported to Stata version 15 for analysis. Chi square test was used for qualitative data to compare results from machine 1 COBAS Taqman (CAP/CTM) and machine 2 (Hologic panther). Pearson correlation test was to determine the relationship between quantitative data generated from COBAS4800 (machine 1) and Hologic panther (machine 2).

### Reporting guidelines

We used the CLSI guidelines, EP15-A3 in our reporting and interpretation of the findings.

## Results

### Qualitative study

Total number of DBS samples retrieved and rerun on COBAS Taqman (CAP/CTM) were 60 of which 30 were negative and 30 were positive. These results were comparable when run on the Hologic Panther machine in which 30 were negative and 30 were positive giving a specificity and sensitivity of 100% and an overall agreement of 100%.

### Quantitative study

#### Accuracy/method correlation

**Fig. 1:**
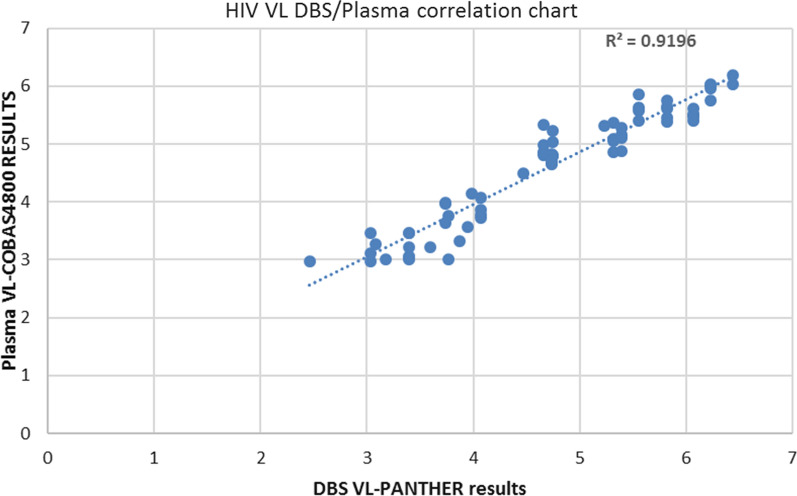
HIV VL DBS/plasma correlation chart

The findings in this study were a positive correlation of 0.96 demonstrating that DBS HIV VL and plasma values correlates well as shown in Fig. 1

### Precision results

Repeatability and within lab mean results for samples of High and low concentrations respectively had CV claims lower than that of the manufactures, indicating the precision claims of the manufacture are true as indicated in Tables 1 and 2.

**Table 1:** High positive precision

Precision studies					
Analyte	Test type	Precision high positive			Acceptability
		Mean	CV (%) claim	LGHL obtained CV (%)	
Viral load (VL) High positive	Repeatability	5.09	2.30	1.116955	Acceptable
	Within Lab		2.30	2.016077	Acceptable

**Table 2:** Low positive precision

Analyte	Test type	Low positive precision			Acceptability
		Mean sample conc.	CV (%) Manf. claim	LCHL obtained CV (%)	
Viral load (VL) Low positive	Repeatability	2.88	4.83	3.50154	Acceptable
	Within Lab		4.83	2.268915	Acceptable

### Linearity results

**Fig. 2:**
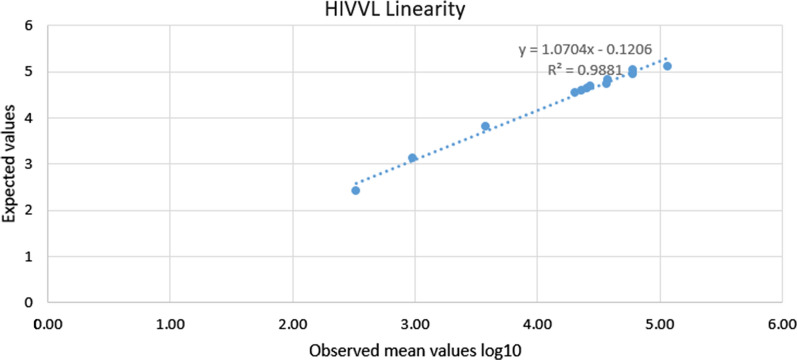
HIV VL linearity chart

All points were within acceptable limits as shown in Fig. 2

## Discussion

Qualitative studies showed the specificity and sensitivity of EID performance of Aptima Quant Dx assay on Hologic panther machine against the gold standard machine COBAS Taqman (CAP/CTM) was 100% with an overall agreement of 100%.

In quantitative studies, correlation studies of the existing testing platforms with Hologic panther machine using the HIV-1 Quant Dx assay were conducted. This was achieved by determining accuracy/method correlation at three levels; Target not detected, below titer**/**log < 2.95 and log > 2.95. TND and log < 2.95 levels were comparable between COBAS4800 and Hologic panther machine. The method correlation was obtained using the correlation coefficient of ± 1, the data obtained from the COBAS C4800 was compared to data generated from the Hologic Panther machine to estimate relationship. Findings in this study correlates with results in a study done in Kenya in which HIV-1 viral load results using DBS samples processed on the Hologic panther were compared with HIV-1 viral load results using plasma samples (from venous blood), showed a positive correlation indicating as plasma values were correlating with the DBS values obtained on the Hologic panther machine [23].

Precision, linearity and uncertainty of measurement were characteristic performance that were analyzed using control material. For precision, it was observed that the manufacturers total CV was greater than the CV repeatability and CV within laboratory obtained by the LUTH during the verification process, thereby being acceptable for both the high positive and low positive controls as shown in Tables 1 and 2.

Linearity studies were determined using a sample with a high concentration and making serial dilutions with reduced concentration and the linearity was at 0.99 regression and all data points were within acceptable range as shown in Fig. 2. Uncertainty of measurement was calculated by using the CLSI guidelines; this was done by using 25 IQC results and using an MU excel calculator to calculate the total MU and the uncertainty of measurement total obtained in this study was at ± 71 copies/ml.

Hologic Panther is an instrument that can be used for running both plasma and DBS samples for viral load testing simultaneously, it is a high throughput machine with random access and runs on a fully automated system. In addition, the Aptima Quant Dx assay used on the Hologic panther is used for both HIV diagnosis and quantification thereby a cost effective option.

## Conclusion

The Hologic Panther machine using Aptima HIV-1 Quant Dx assay with DBS sample protocol can be used for both EID DBS and HIV VL DBS. Findings demonstrated a high sensitive, specific and precise and correlates well with plasma values. Therefore, DBS as a sample type can be used as an alternative for the estimation of HIV-1 viral load and EID diagnosis on the Hologic panther machine.

### Limitations

The use of this assay is limited to personnel who have been trained in the use of the Hologic Panther operation procedure. This assay has been validated for use with LabMate EID DBS collection Bundle K50SUKEID.

## Data Availability

All data generated or analyzed during this study are included in this published Article. For other data, these may be requested through the corresponding Author.

## Abbreviations

EID: Early infant diagnosis
DBS: Dried blood spot
LUTH: Livingstone university teaching hospital
CLSI: Clinical laboratory standards institute
HIV: Human immunodeficiency virus
VL: Viral load
MU: Measurement of uncertainty

## Availability of data and materials

All data generated or analyzed during this study are included in this published Article. For other data, these may be requested through the corresponding author.
